# Supplementary material for: Flow similarity model predicts allometric relationships among Acer platanoides L. branches
Source: PLoS One. 2025 Aug 14;20(8):e0320718. doi: 10.1371/journal.pone.0320718 (PMC12352853; doi:10.1371/journal.pone.0320718)
Supplement: Attachment — Submitted filename: Plos One Second Response.docx [file pone.0320718.s003.docx]

**Second response to reviewers’ comments**

**Thanks again to the handling editor and reviewers for their comments on our manuscript. There were two follow up comments which we address below.**

Reviewer #1: I am largely satisfied with the authors’ responses to my comments on a previous version of this manuscript.

However, I believe that the authors have misunderstood the paper by Smith (2009) regarding the use of OLS versus RMA regression. In their response to one of my previous comments they state: “Smith says (2nd paragraph of first page), “In contrast OLS is indicated when the values for the X-axis are known without error (i.e., all the error can be attributed to the Y-axis measurements).””. Note that this is not Smith’s view but a view that Smith says many investigators use to justify using RMA over OLS. Smith says that this should not be the only criterion. He argues for considering whether the scaling relationship is the same regardless of which variable is the Y versus X. If so, use RMA. If not use OLS. In any case, others point out that even when the X variable has measurement error, OLS should be preferred over RMA if the Y variable is measured with three times more error than that of the X variable (e.g., McArdle 1988; White 2011; Kilmer & Rodríguez 2017). This is because RMA assumes that the Y and X errors are equal (Smith 2009). Kilmer & Rodríguez (2017) also argue that OLS should be preferred over RMA when the measurement errors for both Y and X are relatively low. I hope that this comment is clearer and more helpful than my previous comment. Please see papers listed below for more information.

Kilmer, J. T., & Rodríguez, R. L. (2017). Ordinary least squares regression is indicated for studies of allometry. J. Evol. Biol. 30, 4-12.

McArdle, B.H. (1988). The structural relationship—Regression in biology. Can. J. Zool. 66, 2329–2339.

White, C.R. (2011). Allometric estimation of metabolic rates in animals. Comp. Biochem. Physiol. A Mol. Integr. Physiol. 158, 346–357.

Doug Glazier

**Thanks for your detailed comments on this Doug. I (Price) have done a pretty deep dive on the issue as well. I agree with everything you’ve said here regarding the choice of SMA vs OLS regression. The quote from Smith did catch my eye, but I see your point that Smith was presenting the prevailing view not his view.**

**One challenge is that the error is often unknown a priori and we make some assumptions (which are usually probably reasonable) about which variables have more relative error. Repeated measures could start to get at this but its not the standard in the field.**

**If I understand your comments correctly, you don’t have an issue with the statistics we used. Your original comment was, “SMA is preferred when the error of the Y and X variables are approximately equal, which is probably true for the present study.” Thus, in your view our stats are fine, you’re just making sure we understand Smith’s viewpoint on the issue which we now do.**

**Thanks for your careful reading of our manuscript!**

Reviewer #2: Thank you, authors, for the revision. Authors have adequately addressed both my major and minor concerns regarding the ms. I hope the authors agree that these revisions have improved the ms.

**They have indeed! Thanks again!**

Comment:

Page 19. Line 153. Cite (by author name and date) the raw data (dataset) properly from the university library.

**We’ve changed the reference as requested!**

Thank you once again.

**Thank you!**

**Charles A. Price, on behalf of my coauthors**
